# Epidemiology and diagnostic of MASLD in Latin America: a yet to be met challenge

**DOI:** 10.3389/fphar.2025.1700072

**Published:** 2026-01-26

**Authors:** Carolina Elizabeth Olaez-Ramos, Karla Lizette Mojica-Zamudio, Ana Sandoval-Rodríguez, Juan Armendáriz-Borunda

**Affiliations:** 1 Department of Molecular Biology and Genomics, Institute for Molecular Biology in Medicine and Gene Therapy, Health Sciences University Center, Universidad de Guadalajara, Guadalajara, Mexico; 2 Doctorado en Ciencias en Biología Molecular en Medicina, Health Sciences University Center, Universidad de Guadalajara, Guadalajara, Mexico; 3 Tecnológico de Monterrey, EMCS, Zapopan, Mexico

**Keywords:** diagnostics, epidemiology, LATAM, MASLD, Mexico

## Abstract

Metabolic dysfunction-associated steatotic liver disease (MASLD) has emerged as a major global health concern, affecting more than one-third of the adult population worldwide. Notably, Latin America exhibits the highest regional prevalence, with recent estimates of 44.4%. This elevated burden is attributed to the convergence of risk factors that render the population particularly vulnerable. These include high rates of obesity and type 2 diabetes *mellitus* (T2DM), widespread adoption of energy-dense and nutrient-poor dietary patterns, low levels of physical activity, increased alcohol consumption, and underlying genetic predispositions. Despite its growing impact, MASLD remains significantly underdiagnosed in this region. This is largely due to the limited access to gold-standard diagnostic modalities, such as liver biopsy and magnetic resonance imaging, which are costly, invasive, and often unavailable in routine clinical settings. The lack of accessible diagnostic infrastructure delays early identification and intervention, contributing to disease progression and an increased healthcare burden. Consequently, there is an urgent need to establish cost-effective, non-invasive screening strategies capable of identifying individuals at risk and facilitating timely clinical decision-making. This review synthesizes the current epidemiological data on MASLD in Mexico and Latin America and examines the most validated diagnostic and screening tools currently available.

## 1 Introduction

MASLD is defined as hepatic steatosis (≥5% of hepatocytes) concurrent with at least one of five cardiometabolic risk factors, including overweight/obesity, hyperglycemia, hypertension, and dyslipidemia. Mild alcohol consumption in patients with MASLD, on a weekly basis of 140–350 g of alcohol for females and 210–420 g for males, respectively. Or average daily alcohol consumption between 20 and 50 g for female and 30–60 g for males lead to suspicion of MetALD (Metabolic abnormalities plus hazardous alcohol intake) (Rinella et al., 2023a; Rinella et al., 2023b; Tacke et al., 2024).

The pathogenesis of MASLD is multifactorial and involves a complex interplay between metabolic, genetic, and environmental factors. The disease originates through a sequence of metabolic dysregulations, primarily initiated by excessive hepatic triglyceride accumulation due to a high-calorie/high-fat diet and impaired fatty acid and glucose homeostasis. Additional factors contributing to MASLD pathophysiology include genetic and epigenetic predispositions and dysregulation of the gut–liver axis (Li et al., 2024).

Currently, MASLD is a growing disease, and its prevalence has increased by more than 50% over the past three decades. Recent epidemiological studies have reported global prevalence rates ranging from 30% to 38% of the global population (Wong et al., 2023; Younossi et al., 2023). In fact, this disease accounts for the highest global burden of chronic liver disease, following a significant worldwide increase, with an average annual growth rate of 1.0% (Paik et al., 2023).

Without intervention, these metabolic alterations may progress to metabolic dysfunction-associated steatohepatitis (MASH), followed by fibrosis, cirrhosis, and hepatocellular carcinoma (HCC). Epidemiological data indicate that 12%–40% of patients with MASLD develop MASH within 8–13 years, while approximately 15% of patients with MASH and early fibrosis progress to cirrhosis in the same period, significantly increasing the likelihood of liver transplantation or liver-related mortality (Lekakis and Papatheodoridis, 2024). Although advances in antiviral therapy have reduced hepatitis B as a leading indication for liver transplantation, the overall demand for transplantation remains high due to the increasing prevalence of metabolic and alcohol-related liver diseases. Recent data show that among adults listed for liver transplantation without HCC (representing 83.5% of all transplant candidates), a combined 41.1% were diagnosed with either alcoholic liver disease (ALD) or MASLD as the underlying etiology (Younossi et al., 2018).

Additionally, MASLD is recognized as a systemic condition with a broad range of extrahepatic associations, beyond metabolic and hepatic dysfunction. In the renal domain, MASLD has been identified as an independent predictor of the development of chronic kidney disease (CKD), with reported prevalence rates among patients with MASLD ranging from 20% to 50% (Younossi et al., 2023). Cardiovascular complications are also notable, as MASLD is associated with an approximately 1.5-fold increased risk of both fatal and non-fatal cardiovascular events over a median follow-up of 6.5 years, and more than 50% of affected individuals have coronary artery stenosis (Adams et al., 2017). Finally, growing evidence links MASLD to an elevated risk of certain malignancies, particularly a nearly 3-fold increase in the risk of developing colorectal adenomas (Miao et al., 2024).

**FIGURE 1:**
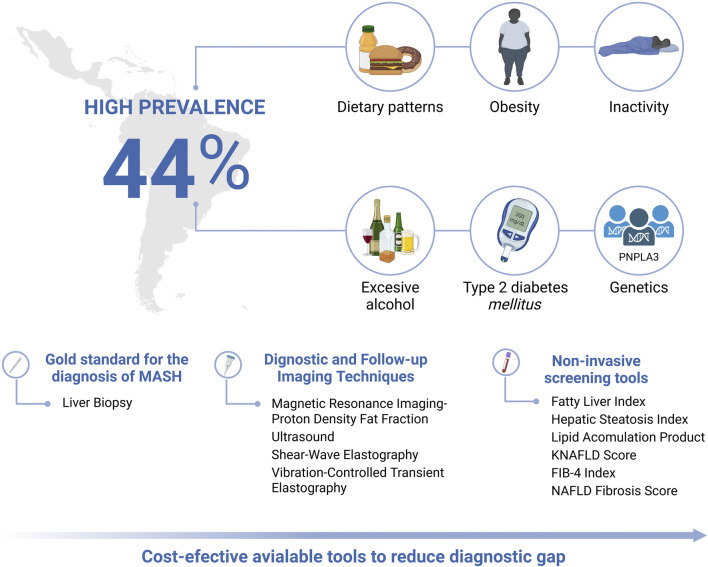
Epidemiological burden, risk factors and diagnostic, follow-up and screening tools for the evaluation of MASLD in Latin America. Created in BioRender. Olaez-Ramos, CE. (2026) https://BioRender.com/mfbzpuv.

In light of this, Latin America, has a population grappling with socioeconomic disparities and significant gaps in access to healthcare, which contribute to poor dietary and physical activity patterns, increased alcohol consumption, and other environmental influences, including exposure to hepatotoxic agents, genetic predispositions, and metabolic dysfunction risk factors (such as obesity and T2DM), all of which contribute to the development and progression of MASLD (Figure 1) (Díaz et al., 2024).

Current diagnostic tools for hepatic steatosis include liver biopsy and transient elastography, which, despite their high accuracy, are limited by their cost and procedural complexity. Liver biopsy carries risks of pain, bleeding, sampling error, observer variability, and adequacy constraints, while transient elastography is limited by high BMI, ascites, probe selection, and variable fibrosis cut-offs across studies. These caveats highlight why non-invasive pathways require local validation and calibration to ensure reliable triage in primary care. Non-invasive screening methods based on clinical, anthropometric, and biochemical parameters offer an alternative for risk estimation (Tacke et al., 2024; Yang et al., 2024).

However, as hepatic steatosis is asymptomatic, it is frequently overlooked by primary care physicians, who often prioritize other obesity-related comorbidities, resulting in a delayed diagnosis. Thus, the implementation of cost-effective predictive tools could facilitate timely risk stratification and improve early detection.

In Latin America, the high prevalence of obesity and its associated comorbidities significantly contribute to the growing burden of MASLD. The true scale of this condition remains underdiagnosed, highlighting the urgent need for improved diagnostic tools to enable its timely detection and effective management. However, the diagnosis and screening of MASLD in Mexico and other Latin American countries face specific challenges. Under current definitions, MASLD diagnosis requires not only the presence of ≥5% hepatic steatosis but also at least one cardiometabolic risk factor, such as obesity, type 2 diabetes, or dyslipidemia. This metabolically anchored framework differs from prior definitions of NAFLD and complicates population-level screening, particularly in settings with limited access to imaging or laboratory resources. Furthermore, metabolic abnormalities are frequently underrecognized in routine clinical care, leading to missed opportunities for early diagnosis. These limitations underscore the need for validated, affordable screening strategies adapted to regional healthcare infrastructures, and for greater integration of metabolic risk profiling into liver disease surveillance protocols (Tacke et al., 2024).

## 2 MASLD: prevalence and complication patterns

To identify relevant epidemiological data on MASLD, we conducted a structured literature search using Google Scholar. In light of the recent nomenclature change for this disease, all historical terminology was considered, employing the search terms “MASLD” OR “MAFLD” OR “NAFLD” AND “prevalence” AND “Latin America” AND “Hispanic.” Although terminology and diagnostic criteria differ, MASLD-defined populations substantially coincide with historically NAFLD-defined groups, showing similar prevalence rates (Kanwal et al., 2024). These findings suggest that results from studies in patients with NAFLD may be extrapolated to those with MASLD. The search strategy was designed to identify studies reporting prevalence estimates and epidemiological patterns among Spanish-speaking populations in Latin America and Hispanic groups in the United States. Studies were included irrespective of publication language, provided that data were derived from robust sources such as national health surveys or population-based cohorts. Only studies that excluded participants with excessive alcohol consumption or with alternative causes of liver disease or hepatic steatosis (e.g., medication-induced injury, viral hepatitis, HIV infection) were included; when this criterion was not met, it is explicitly stated (Table 1).

**TABLE 1:** Summary of MASLD/NAFLD prevalence estimates in Hispanic populations and Latin American countries.

Country/Population	Prevalence (%)	Diagnostic method	Reference
Latin America	44.4%	Meta-analysis 3 studies (n = 5,875) Using diverse imaging modalities (ultrasound, magnetic resonance imaging and magnetic resonance spectroscopy, elastography, and computed tomography, as well as serum-based indices such as the fatty liver index, the ultrasound-fatty liver index, and the hepatic steatosis index)*	Younossi et al. (2023)
U.S. Hispanics	41%	Meta-analysis 22 studies (n = 62,072) Age 44.6–60 years Using diverse imaging modalities (magnetic resonance imaging, elastography, liver-spleen ratio, Aminotransferases and MASLD liver fat score and liver biopsy)*	Tesfai et al. (2025)
U.S. Hispanics	45.5%	Cohort study n = 15, 216 from the hispanic community health study/study of Latinos (HCHS/SOL) Age 18–74 years MASLD was defined by the presence of steatosis using the FLI (values ≥60) and at least one metabolic risk abnormality according to current international guidelines.***	Trejo et al. (2025)
Chile	39.4% 27.2%	Cross-sectional study n = 2,744 Age 21–75 years Fatty Liver Index (values ≥60 were considered indicative of NAFLD) Lipid Accumulation Product (LAP ≥4.4 severe fat accumulation)	Pettinelli et al. (2023)
Argentina	37.2%	Cross-sectional study n = 933 The Median (IQR) age was 44 (29–59) years Abdominal Ultrasound	Villamil et al. (2023)
Mexico	49.6%	Cross-sectional study n = 3,357 participants (in 5 Mexican states) Mean age 47 ± 12 years Ultrasound (Hepatic steatosis was defined as present when ultrasound showed steatosis grade ≥1) + clinical screening score (MAFLD-S) (optimal cut-off of 47.67).**	Ruiz-Manríquez et al. (2022)
Mexican Americans	46.7%	Examination Survey (NHANES 2017–2018) n = 1710 participants Mean age 46.4 years VCTE (CAP ≥274 dB/m)	Ciardullo and Perseghin (2021)
Prevalence of MASLD in Latin America: Estimates derived from GBD 2019			
Argentina	8.02%	Age-standardized modeling: Data derived from the Global Burden of Disease (GBD) 2019 model-based estimates, using standardized case definitions and methods to enable comparison across regions and countries	Díaz et al. (2024)
Chile	9.43%		
Mexico	17%		

The table includes the maximum available information on population characteristics (sample size, age range, mean age and/or age distribution); when these data were not reported in the original articles, this is indicated accordingly. *=Cut-off points not reported. **=This study included subjects with alternative causes of liver disease or hepatic steatosis (medication-induced injury, viral hepatitis, HIV, infection, among others) ***=This study included subjects with alternative causes of liver disease or hepatic steatosis (excessive añcohol consumption, medication-induced injury, viral hepatitis, HIV, infection, among others).

Globally, Latin America has been identified as the region with the highest prevalence of MASLD, reaching 44.4% according to population-based estimates derived from studies conducted between 1990 and 2019. These findings stem from a PRISMA-based systematic review encompassing 92 eligible studies and over 9.3 million individuals, where MASLD/NAFLD diagnoses were established using various imaging modalities, such as ultrasound, MRI, MR spectroscopy, elastography, and CT as well as validated serum-based indices (e.g., fatty liver index, ultrasound-fatty liver index, hepatic steatosis index) (Younossi et al., 2023).

Consistently, among Hispanic adults in the United States, a recent meta-analysis involving over 62,000 individuals reported a pooled MASLD prevalence of 41% (95% CI: 30%–52%), with a significantly higher relative risk compared to non-Hispanic populations (RR 1.50; 95% CI: 1.32–1.69) (Tesfai et al., 2025). These finding underscores persistent ethnic disparities and the urgent need for culturally and demographically tailored interventions.

Within Latin America, countries such as Chile, Argentina, and Mexico have emerged as key reference points due to the availability of population-based data and high prevalence rates. The most recent findings from these countries are summarized below.

In Chile, estimates from the Global Burden of Disease (GBD) study report a MASLD prevalence of 9.43% in 2019 (Díaz et al., 2024). These GBD estimates integrate data from censuses, health surveys, vital statistics, and disease registries from 204 countries. However, more detailed, population-based analyses using the 2016–2017 National Health Survey revealed higher estimates: 39.4% (95% CI: 36.2–42.8) using the Fatty Liver Index (FLI) and 27.2% (95% CI: 24.2–30.4) based on the Lipid Accumulation Product (LAP) (Pettinelli et al., 2023).

In Argentina, a study conducted in a suburban cohort of 993 adults estimated a NAFLD prevalence of 37.2% based on abdominal ultrasound and standard diagnostic criteria (Villamil et al., 2023). In contrast, the GBD study reports a significantly lower prevalence of 8.02%, reflecting the discrepancy between localized diagnostic efforts and global modeling estimates (Díaz et al., 2024).

In Mexico, the GBD study reported a prevalence of 17% (Díaz et al., 2024). More recently, a 2022 cross-sectional study across five Mexican states involving 3,357 adults estimated a MASLD prevalence of 49.6%, based on liver ultrasound and a clinical screening score (Ruiz-Manriquez et al., 2022).

Among Mexican Americans living in the United States, data from the 2017–2018 National Health and Nutrition Examination Survey (NHANES) indicated a hepatic steatosis prevalence of 46.7%, using vibration-controlled transient elastography (VCTE) and a controlled attenuation parameter (CAP ≥274 dB/m) a validated threshold for the detection of steatosis. This subgroup presented the highest prevalence among all ethnic groups assessed (Ciardullo and Perseghin, 2021).

In parallel, the GBD database reported that Mexico had one of the highest death rates from MASLD globally in 2021, with 7.24 deaths per 100,000 inhabitants (Huang et al., 2025). Across Latin America, liver-related mortality attributed to MASLD exceeds that observed in regions such as Southeast Asia, East Asia, Oceania, North Africa, the Middle East, and Sub-Saharan Africa. This elevated mortality burden is closely associated with the high prevalence of obesity, type 2 diabetes mellitus (T2DM), and metabolic dysfunction (Younossi et al., 2023). Moreover, the presence of multiple metabolic abnormalities substantially increases the risk of mortality in individuals with MASLD, reinforcing the need for early identification and management of cardiometabolic risk factors (Cusi et al., 2025).

Despite these insights, up-to-date and region-specific epidemiological estimates remain scarce for many Latin American countries. This data gap limits accurate burden estimation, hinders the design of effective public health policies, and complicates the establishment of diagnostic thresholds suited to the regional context. To address this, Table 1 consolidates all currently available MASLD prevalence data, detailing population characteristics, diagnostic methods, and sources, and providing a clearer overview of the burden across the region.

## 3 MASLD risk factors in Latin America/México

Latin America comprises 20 nations, accounting for approximately 8% of the world’s population and displaying considerable ethnic and genetic diversity (CEPAL, 2022). Numerous factors influence the prevalence and impact of liver disease in this region. Key among these are social determinants of health, such as socioeconomic status, educational attainment, food insecurity, and urban infrastructure, which significantly shape dietary habits, physical activity levels, alcohol consumption, and healthcare accessibility. These factors are closely associated with the incidence and progression of liver-related conditions. Additionally, genetic predispositions and metabolic risk factors, including being overweight, obesity, and T2DM, further contribute to the regional burden of liver disease, affecting both its prevalence and clinical outcomes (Díaz et al., 2024).

### 3.1 Obesity

According to a meta-analysis encompassing 86 studies with a total sample of 8,515,431 individuals from 22 countries, obesity remains highly prevalent among those with MASLD, affecting 51.34% of cases (Younossi et al., 2016). Importantly, MASLD is not confined to individuals with excess body weight. Evidence from a cross-sectional observational study conducted in a tertiary care center in Eastern Europe demonstrated that the disease can also occur in individuals with a normal body mass index, a phenotype commonly referred to as “lean MASLD.” In a cohort of 1,438 adult patients assessed using VCTE with the controlled attenuation parameter, MASLD was detected in 19.28% of normal-weight individuals (Rotaru et al., 2025).

Strong evidence indicates that obesity increases the risk of developing MASLD, with an estimated odds ratio of 4.6 (Finucane et al., 2011). This association is primarily attributed to the excessive accumulation of adipose tissue, which contributes to hepatic lipid overload and disrupts several pathways involved in metabolic energy homeostasis. Additionally, obesity promotes a state of chronic subclinical inflammation in both adipose tissue and the liver while inducing insulin resistance across key metabolic organs, particularly the liver. Beyond insulin resistance, the dysregulated release of adipokines and proinflammatory cytokines from inflamed adipose tissue, along with aberrant lipid signaling, further exacerbates hepatic dysfunction (Stefan et al., 2025).

In Latin America, data from 2025 indicate that obesity prevalence in the general population ranges from 24% to 44%, with the highest rates observed in Argentina (39%), Chile (42%), Mexico (39%), and Puerto Rico (44%). This data emphasize the considerable burden of MASLD across the region (World Obesity Federation, 2025).

### 3.2 Type 2 diabetes *mellitus*

MASLD is an independent and clinically relevant risk factor for the development of T2DM. In Mexico, the prevalence of prediabetes is estimated to be 22.1%, whereas the prevalence of diagnosed and undiagnosed T2DM is 12.6% and 5.8%, respectively (total T2DM prevalence of 18.3%) (Basto-Abreu et al., 2023). These high rates of glucose metabolism disorders are particularly relevant, given that approximately 65.1% of individuals with T2DM have concomitant MASLD. This finding derives from a study conducted in the United States, in which 41% of the sample population were Hispanic individuals (Ajmera et al., 2023). Notably, MASLD is also prevalent in the earlier stages of metabolic disease, with reported rates ranging from 37% to 50% among individuals with prediabetes (Cusi et al., 2025). This epidemiological overlap underscores the potential magnitude of MASLD in the Mexican population, particularly in the context of the growing burden of metabolic disorders.

The relationship between MASLD and T2DM is clearly bidirectional, driven by shared pathophysiological mechanisms, particularly insulin resistance (IR). On one hand, evidence from large-scale prospective cohort studies, including over 500,000 individuals with a median follow-up of 5 years, has shown that MASLD is associated with an approximately 2.19-fold increased risk of developing T2DM (95% CI: 1.93–2.48) (Targher et al., 2021). This progression is largely explained by the hepatic accumulation of lipids, especially diacylglycerols (DAG), which activate protein kinase Cε (PKCε) and impair insulin receptor signaling, notably through inhibition of the PI3K/Akt pathway. This promotes gluconeogenesis, reduces glycogen synthesis, and enhances *de novo* lipogenesis, contributing to systemic metabolic dysregulation. MASLD is also frequently accompanied by atherogenic dyslipidemia, characterized by elevated levels of very low-density lipoproteins (VLDL) and small dense LDL particles, alterations commonly observed in individuals with T2DM. On the other hand, T2DM can initiate or exacerbate MASLD through increased lipolysis in dysfunctional adipose tissue, reduced adiponectin levels, elevated free fatty acids, and activation of proinflammatory pathways. Additionally, ceramide accumulation, particularly ceramide C16, suppresses Akt activity and worsens hepatic insulin resistance, promoting lipotoxicity, hepatocellular injury, and progression to steatohepatitis and fibrosis. This bidirectional interaction highlights the need for integrated clinical strategies targeting both conditions simultaneously (Ntikoudi et al., 2025).

### 3.3 Dietary patterns

Growing evidence suggests that food insecurity significantly contributes to the rising prevalence of MASLD by aggravating key metabolic risk factors, particularly obesity and T2DM. Food insecurity, characterized by the unreliable access to sufficient, safe, and nutritious food necessary for an active and healthy life, encompasses deficits in both the quantity and quality of food, constrained accessibility to nutritious dietary options, and insufficient availability of safe drinking water (FAO et al., 2023).

Epidemiological data highlight a striking disparity in the prevalence of MASLD between regions with severe food insecurity (26.73%) and those with minimal food insecurity (18.87%). This disparity might be driven by the dual influence of food insecurity, which often necessitates a reliance on energy-dense, nutrient-poor, and ultra-processed foods, thereby exacerbating metabolic risk factors for MASLD. To establish this association, Younossi et al. (2024) conducted an ecological analysis that correlated adult MASLD prevalence estimates from the Global Burden of Disease (GBD) 2021 study with independent food security indicators from FAOSTAT for the same year. Together, these databases encompassed data from over 245 countries worldwide. These indicators, which included the prevalence of undernourishment and the cost of a healthy diet, were measured using the Food Insecurity Experience Scale Survey Module (FIES-SM) to capture both moderate and severe forms of food insecurity (Younossi et al., 2024).

This pattern aligns with a 2018 global diet quality study spanning 185 countries, which employed the validated Alternative Healthy Eating Index (AHEI; 0–100 scale), identified Latin America as the region with the lowest dietary quality. The mean AHEI score for Latin America was 30.3 (95% UI: 28.7–32.2), which is substantially lower than the global average of 40.3 (95% UI: 39.4–41.3) (Miller et al., 2022).

Like many Western nations, Latin American countries are marked by the widespread consumption of diets high in calories and saturated fats. Mexico, Argentina, and Chile are among the top five global consumers of sugar-sweetened beverages. Across the region, added sugar intake exceeds the limit recommended by the World Health Organization (WHO) by threefold (Popkin and Reardon, 2018). Furthermore, findings from the Latin American Study of Nutrition and Health (ELANS), a cross-national investigation encompassing Argentina, Brazil, Chile, Colombia, Costa Rica, Ecuador, Peru, and Venezuela, revealed that fewer than 3.5% of the 9,218 participants achieved the recommended intake levels of key nutrient-dense foods, including vegetables, nuts, whole grains, fish, and yogurt (Fisberg et al., 2016). This disparity is particularly evident in Mexico, where only 49% of the population consumes vegetables compared to 76% who regularly consume sugar-sweetened beverages (Gaona-Pineda et al., 2023).

### 3.4 Genetic influence

As previously discussed, the pathophysiology of MASLD encompasses a diverse array of complex mechanisms involving both environmental and genetic factors that influence the susceptibility and progression of the disease (Eslam et al., 2018; Cotter and Rinella, 2020; Souza et al., 2024). Among these, the single-nucleotide polymorphism rs738409 (C>G) in the PNPLA3 gene, which encodes the I148M variant, has been strongly associated with disease development (Mazo et al., 2019).

This variant impairs lipolysis due to the loss of enzymatic function. Specifically, the I148M substitution promotes the accumulation of triglycerides and retinyl esters within lipid droplets in hepatocytes and hepatic stellate cells (Pirazzi et al., 2014). The mechanism of damage involves the accumulation of the mutated 148M variant on lipid droplets, where it evades proteasomal degradation and suppresses the activity of other lipases, particularly ATGL (Basuray et al., 2017). Consequently, these metabolic disturbances contribute to increased hepatic fat deposition and exacerbate hepatic inflammation (Romeo et al., 2008).

Furthermore, studies have demonstrated that patients carrying the PNPLA3 GG genotype have a significantly higher risk of overall mortality, liver-related events, cirrhosis, and liver-related mortality than those with the CC genotype (Souza et al., 2024). Moreover, the PNPLA3 GG genotype showed association with FIB-4 (β = 0.07; 95% CI: 0.03–0.11) (Kallwitz et al., 2019). Additionally, evidence suggests that Hispanic individuals with the PNPLA3 GG genotype are at an increased risk of significant fibrosis, with this risk becoming apparent by age 40 and progressively worsening with advancing age (Díaz et al., 2024).

A comprehensive meta-analysis of 109 studies involving 118,302 individuals with MASLD revealed intriguing patterns in the distribution of the PNPLA3 rs738409 G allele. The analysis demonstrated an overall minor allele frequency [MAF(G)] of 0.45 (95% CI: 0.43–0.48). Geographical analysis revealed striking regional variations in allele distribution. Latin American populations exhibited the highest MAF(G) values globally, whereas European cohorts showed the lowest frequencies. Particularly noteworthy were the findings from Mexico, which reported a MAF(G) af of 0.69 (95% CI: 0.66–0.72), the highest observed in the analysis (Souza et al., 2024). This corresponds to a genotype frequency of 52% in the Mexican population with suspected MASLD, defined by elevated ALT and AST levels according to the authors. Moreover, the PNPLA3 G allele was associated with suspected NAFLD among Cubans (OR 1.8; 95% CI 1.5–2.2), Central Americans (OR 1.6; 95% CI 1.3–2.0), and South Americans (OR 1.4; 95% CI 1.0–1.9). In contrast, no association was observed in individuals of Puerto Rican or Dominican ancestry (Kallwitz et al., 2019).

Other Latin American countries also exhibited elevated frequencies: Argentina recorded an MAF(G) of 0.63 (95% CI: 0.56–0.70), while Brazil reported 0.44 (95% CI: 0.40–0.47) (Souza et al., 2024). Consistent with these findings, a study of Latin American populations (including subjects from Argentina, Brazil, and Chile) reported the following PNPLA3 genotype distribution: C/C, 35.7%; C/G, 42.6%; and G/G, 21.6% (Díaz et al., 2025a). Notably, some studies have documented even higher frequencies in Peru (72%) and Guatemala (69%) (Lazo et al., 2022; Kozlitina and Sookoian, 2024).

Additionally, several other genetic variants contribute to liver disease development. Among them, the transmembrane 6 superfamily member 2 (TM6SF2) gene plays a key role in the hepatic secretion of very low-density lipoproteins (VLDL) by facilitating the loading of triglycerides onto apolipoprotein B100. The rs58542926 C>T polymorphism results in a glutamate-to-lysine substitution at position 167 (E167K), leading to the loss of TM6SF2 function. This impaired function promotes hepatic triglyceride accumulation while reducing the circulating lipoprotein levels (Kozlitina and Sookoian, 2024). Importantly, this variant is significantly associated with MASLD, disease progression, and the presence of advanced steatosis and fibrosis, as supported by a meta-analysis encompassing data from 123,800 individuals across 44 studies (Li X. Y. et al., 2022).

However, the prevalence of this polymorphism is relatively low. In a cohort of 570 individuals with MASLD in the United States, which included 34.2% of participants of Hispanic descent, the genotype distribution was reported as 84.9% C/C, 13.5% C/T, and 1.6% T/T (Díaz et al., 2025b). An even lower frequency (approximately 5%) was observed in a larger study involving 16,415 Hispanic/Latino individuals recruited from four U.S. communities. Specifically, MAF was higher in Puerto Ricans (7.0%, *p <* 0.05) and Cubans (6.3%, *p* < 0.05) compared with Mexicans (6.2%). These findings suggest subtle differences in allele distribution across Hispanic subgroups (Kallwitz et al., 2019).

Although the literature clearly associates these genetic variants with increased risk of disease, most studies in Latin American populations have been conducted in subgroups of individuals with Latino ancestry residing in the United States. This underscores the urgent need for extensive studies directly in Latin American populations, particularly among subjects diagnosed with MASLD.

## 4 Barriers to effective management of MASLD in Latin America

In Latin America, both physician and patient awareness of MASLD is limited, despite the high burden of metabolic risk factors in the region. This lack of awareness contributes to poor recognition of the disease, underdiagnosis, and delayed initiation of appropriate treatment, ultimately compromising epidemiological data (Arab et al., 2021; Díaz et al., 2024).

Addressing the growing burden of liver disease in Latin America requires overcoming multiple barriers, particularly improving the identification of individuals at risk of developing or progressing to advanced stages of MASLD. Implementing simple, cost-effective strategies for stratification, such as the use of anthropometric, biochemical, and other accessible clinical indices, should be prioritized. This is especially relevant in primary care settings and among high-risk populations, including individuals with obesity and T2DM (Rinella et al., 2023a; Rinella et al., 2023b; Tacke et al., 2024; Diaz et al., 2025).

## 5 MASLD diagnosis in Mexico and LATAM: barriers and opportunities for improvement

MASLD can be diagnosed using both invasive and non-invasive methods (Table 2). Non-invasive methods are increasingly favored because of their accessibility and lower risk, with some models showing strong diagnostic accuracy comparable to that of biopsy. However, liver biopsy remains the gold standard for diagnosis.

**TABLE 2:** Comparative overview of non-invasive tools for diagnosing MASLD in LATAM.

Tool	Cost	Availability	Operator dependence	BMI effects	Probe selection
Clinical risk scores (FIB-4, NFS, HSI, FLI)	Very low	Very high (primary care)	Very low	Minimal; may lose specificity in elderly	NA
Conventional ultrasound (B-mode steatosis grading)	Low	High (primary & secondary)	High (image quality, reader expertise)	Reduced sensitivity in obesity	Standard abdominal probe
Transient elastography with CAP (FibroScan)	Moderate	Moderate–low (mostly specialty/tertiary)	Low–moderate	CAP and stiffness affected by obesity	M/XL probe choice critical
Point shear wave/2D-SWE elastography (US-based)	Moderate	Moderate (equipment variability)	Moderate–high	Attenuation and depth limits with obesity	High-frequency abdominal probes
Serum fibrosis panels (e.g., ELF)	Moderate	Low–moderate (urban specialty labs)	Very low	Minimal	NA
MRI-PDFF	High	Low (tertiary centers)	Low	Minimal	NA
CT (non-contrast)	Moderate	Moderate	Low	Less accurate for mild steatosis	NA

2D-SWE, two-dimensional shear wave elastography; CAP, controlled attenuation parameter; CT, computed tomography; ELF, enhanced liver fibrosis; FIB-4, fibrosis-4 index; FLI, fatty liver index; HSI, hepatic steatosis index; MRI-PDFF, magnetic resonance imaging–proton density fat fraction; NA, non applicable; NFS, NAFLD fibrosis score; SWE, shear wave elastography; US, ultrasound.

### 5.1 Invasive methods

#### 5.1.1 Liver biopsy

Liver biopsy is a medical procedure in which a small sample of liver tissue is taken for microscopic examination for detailed histological analysis, aiding in the diagnosis of liver diseases, assessing and staging the severity of liver damage, guiding treatment decisions, evaluating treatment response, and monitoring liver disease progression or post-transplant liver health. Liver biopsy is particularly important for staging fibrosis. The fibrosis stage is a strong predictor of liver-related mortality and morbidity. Early detection and staging of fibrosis are vital because mild-to-moderate fibrosis can be reversed with appropriate treatment. Advanced stages, such as cirrhosis, are associated with a higher risk of complications such as portal hypertension, variceal bleeding, and hepatocarcinoma.

Liver biopsy has evolved to be less invasive and requires less post-procedure care. However, it remains costly because of the specialized personnel and equipment required for precise follow-up. Several liver biopsy methods, including percutaneous, transvenous, laparoscopic, and plugged biopsy techniques, can be used. Each method has specific indications based on patient factors (e.g., risk of bleeding or need for a targeted biopsy). Percutaneous Liver Biopsy is the most common method and is guided by ultrasound or CT imaging to ensure accuracy. It is minimally invasive but carries a small risk of bleeding and pain. Transjugular Liver Biopsy is used for patients with bleeding disorders or excessive ascites, as it reduces the bleeding risk compared to percutaneous biopsy. For this procedure, a catheter was inserted into the jugular vein and guided to the liver to collect the sample. Laparoscopic Liver Biopsy is usually performed during surgery using a laparoscope inserted into the abdomen that allows direct visualization of the liver and targeted sampling. It is mostly used when other methods are not feasible or when additional procedures are required. Liver tissue obtained from the biopsy is usually stained with Hematoxilin & Eosin, and Masson’s trichrome for histological analysis.

Liver biopsy remains the gold standard for diagnosing various liver disorders, such as MASLD, autoimmune liver diseases, chronic viral hepatitis, cirrhosis, liver cancer, liver storage diseases, unexplained hepatomegaly, and drug-induced liver injury. Liver biopsy is typically considered when there is a need to confirm advanced fibrosis, discordance in non-invasive tests, clinical trial endpoints, or concerns when alternative etiologies of liver disease are suspected.

Particularly in MASLD, liver biopsy also serves as a prognostic tool because the presence of fibrosis or cirrhosis has significant prognostic implications. In MASLD, liver biopsy is crucial for accurately staging fibrosis, which is a key factor in determining prognosis and guiding treatment. It also helps distinguish between MASLD and more severe conditions, such as MASH. MASLD encompasses both steatosis without MASH. MASH is characterized by steatosis, ballooning degeneration of hepatocytes, and lobular inflammation, which can only be detected in liver biopsy. It is estimated that up to one in four patients with MASLD have MASH. Differentiating between these conditions is important because they have different prognoses and treatment strategies. Once MASH develops, it can advance to fibrosis, cirrhosis, and HCC.

##### 5.1.1.1 NAFLD activity score (NAS)

In 2005, the Pathology Committee of the NASH Clinical Research Network designed and validated a histological scoring system using liver biopsies stained with H&E and Masson’s trichrome for the evaluation of the full spectrum of lesions -in those times called- of NAFLD and proposed the NAFLD activity score (NAS) for use in clinical trials. The scoring system comprises 14 histological features. Four to be evaluated semi-quantitatively: steatosis (0–3), lobular inflammation (0–2), hepatocellular ballooning (0–2), and fibrosis (0–4). Nine features were reported as present or absent: microvesicular steatosis, microgranulomas, large lipogranulomas, portal inflammation, acidophil bodies, pigmented macrophages, megamitochondria, Mallory´s hyaline, and glycogenated nuclei. MASH using this scale can be defined as NAS ≥ 5 (Kleiner et al., 2005).

Although liver biopsy is not obligatory for diagnosing MASLD, providing histopathological features will accurately assess fibrosis progression, which is a significant risk factor for hepatocellular carcinoma and mortality. While non-invasive tests are improving, they may not always be accurate in detecting early fibrosis or distinguishing between MASLD and MASH. Therefore, in specific situations, particularly when non-invasive tests are inconclusive or when a more accurate assessment is needed, liver biopsy remains an important tool for managing MASLD. However, it is unlikely that there will be enough medical manpower available for routine screening of MASLD using liver biopsy. However, in clinical research settings, liver biopsy is often used as a primary endpoint to assess the effectiveness of new treatments for MASLD and MASH, and biopsy-confirmed fibrosis remains the gold standard for predicting liver-related complications and mortality (Hudson et al., 2024).

##### 5.1.1.2 Liver biopsy limitations

Liver biopsy limitations include procedure accessibility since it requires trained personnel and specialized equipment, its invasive nature and risk of complications like bleeding and pain and elevated cost (Gonzalez et al., 2013). However, other concerns are present, such as significant sampling error due to the small tissue sample being taken, which can be potentially unrepresentative of the organ and can lead to inaccurate diagnosis. Since only 1/50,000 of the whole liver tissue is sampled during a liver biopsy a sampling error is of concern (Sumida et al., 2014). Additionally, insufficient tissue can be obtained, making it difficult to accurately evaluate liver disease. To prevent sampling errors, it is essential to collect a sufficient amount of tissue; an adequate biopsy specimen is defined as having a length of no less than 10 mm and no less than six portal tracts (Bell, 2020). An accurate diagnosis of MASH depends on the length of the specimens, with a necessary length of 15–16 mm or longer to accurately evaluate fibrosis (Clin Gastroenterol Hepatol. 2009; 7:481–486). Therefore, the collection of two or more samples with sufficient length may be required. Inconsistency has been reported in fat content and fibrosis between biopsied specimens from the left and right lobes, differing by one or more stages in 30% of patients (Merriman et al., 2006) (Obes Surg. 2005; 15:497–501).

Observer variability in the histopathological interpretation of liver tissue remains a significant limitation, contributing to diagnostic inconsistencies, particularly in conditions such as MAFLD. While inter- and intra-observer assessments of steatosis and fibrosis have demonstrated high concordance, evaluations of inflammatory activity exhibit substantial inconsistency (Mod Pathol. 1998; 11:560–565). To mitigate this issue, standardized and reproducible scoring systems such as NAS are commonly employed. NAS offers several advantages: it does not require special staining, is applicable to pediatric cases of MASH, and facilitates the evaluation of therapeutic efficacy in clinical trials. Nonetheless, discrepancies in pathological diagnoses using NAS have been reported between general pathologists and those with hepatology specialization (J Clin Gastroenterol. 2011; 45:55–58). Moreover, NAS is limited in its diagnostic utility for MASH in patients who, following treatment, exhibit resolution of steatosis and inflammation but retain residual fibrosis.

##### 5.1.1.3 Liver biopsy use in Mexico

The risk factors for SLD are frequent in the Mexican population, and the available studies to determine the epidemiology of MASLD in Mexico are scarce and have been reported using non-invasive tests (León-Plascencia et al., 2021; Bernal-Reyes et al., 2023). It is undeniable that Mexico and most countries in LATAM do not have the infrastructure and economic possibility to use liver biopsy as a screening tool for MALSD. Social health systems are surpassed, and in the second or third grade of medical attention, MASLD is diagnosed using non-invasive tests such as ultrasound and liver enzyme determinations, and in a few cases, elastography. Most liver biopsies performed in the country are performed in private medical practice, where the patient´s resources cover the cost of the procedure. Public health institutions can only afford liver biopsies in limited cases or when involved in clinical trials (López-Velázquez et al., 2014).

### 5.2 Non-invasive methods

As liver fibrosis and inflammatory activity are the strongest predictors of liver-related morbidity and mortality in MASLD, the development of non-invasive approaches for evaluating hepatic steatosis, fibrosis, and inflammation has become a clinical imperative. Accordingly, a range of non-invasive tests (NITs) targeting these parameters has been introduced over recent decades to enhance diagnostic accuracy and reduce reliance on liver biopsy.

#### 5.2.1 Imaging techniques

##### 5.2.1.1 Ultrasound

Ultrasound is the first-choice imaging tool for screening and diagnosing MASLD because it is safe, widely available, and low-cost. While conventional B-mode ultrasound can subjectively grade the severity of fat in the liver by looking for signs such as increased brightness and loss of detail, its sensitivity for steatosis detection declines in individuals with high BMI, for mild steatosis (<20% fat), when alcohol-induced steatosis coexists, and in advanced fibrosis, potentially delaying MASLD recognition (Pierce and Samir, 2022; Frączek et al., 2025). Ultrasound is widely used in routine clinical practice, including ambulatory settings; however, a negative ultrasound does not exclude NASH or fibrosis, although it may provide relevant information when cirrhotic morphology or signs of portal hypertension are observed. Despite these limitations, ultrasound remains a practical tool for the semi-quantitative assessment of steatosis severity (Rinella et al., 2023a; Rinella et al., 2023b). To address US limitations, newer techniques such as ultrasound (US)-based liver elastography now quantify tissue stiffness as a surrogate marker of liver fibrosis, measuring fat, inflammation, and fibrosis more objectively. Ultrasound elastography can be divided into three approaches: semi-quantitative strain elastography, shear wave elastography (SWE), and vibration-controlled transient elastography (VCTE).

##### 5.2.1.2 Quantitative US-methods

These quantitative US methods include the measurement of the attenuation coefficient (AC), backscatter coefficient (BSC), and sound speed. Attenuation refers to the energy loss of the US wave resulting from the combination of absorption, reflection, refraction, scattering, and diffusion when it passes through an organ. AC is expressed in dB/cm/MHz, and in the liver, it is expected to range from 0.43 to 1.26 dB/cm/MHz. Modern ultrasound platforms achieve an AUC >0.85 for distinguishing all grades of hepatic steatosis. Accordingly, the incorporation of AC into routine medical protocols, including ambulatory screening strategies, has been recommended, particularly since its diagnostic value appears greater in cases of advanced or severe steatosis (Wu et al., 2025).

Backscatter refers to the reflection of US compression waves by the tissue. Experimental studies have demonstrated a positive correlation between backscatter and hepatic steatosis. The BSC is defined as the differential scattering cross-section per unit volume in the 180° direction. The speed of sound is expressed in m/s. Fat accumulation in the liver leads to a significant decrease in the speed of sound (1,423–1,567 m/s) compared to that in a healthy human liver (1,538–1,588 m/s). Thus, speed of sound has a strong negative correlation with hepatic fat content (Rou, 2024).

###### 5.2.1.2.1 Semi-quantitative strain elastography

This ultrasound-based technique measures tissue stiffness by evaluating tissue deformation under pressure, providing a relative measure of stiffness, rather than an exact value. The strain ratio (SR) is calculated by comparing the deformation in a lesion or fat accumulation to the deformation in normal surrounding tissue. Softer tissues deform more (higher strain), whereas harder tissues deform less (lower strain). The selection of interest (ROIs) for the strain ratio calculation is subjective, as different performers may choose different ROIs, influencing the results. It is reported to be more objective than purely qualitative methods but less precise than quantitative methods, such as shear wave elastography (Onur et al., 2012).

###### 5.2.1.2.2 Shear-wave Elastography (SWE)

Shear-Wave Elastography (SWE) is an Acoustic Radiation Force Impulse (ARFI)-imaging ultrasound-based technique. SWE uses focused ultrasound pulses to generate localized shear waves; it captures the propagation speed of the shear wave (m/s) within a region of interest (ROI) and derives tissue elasticity (kPa). It quantitatively assesses the elasticity and stiffness of the tissue. The shear-wave velocity is related to the elastic modulus of the tissue: in harder tissue, the shear wave propagates faster, whereas in softer tissue, it propagates slower. Higher shear wave speeds indicate increased stiffness and fibrosis. Existing clinical standards stratify the risk of liver fibrosis based on a composite of SWE measurements and clinical evaluation. SWE has three modalities: Point-SWE (p-SWE), which is the point-shaped measurement of wave velocity in a single region of interest (ROI) using acoustic force impulses (ARFI). It provides numerical values ​​of velocity (m/s) or modulus (kPa) in real time. Two-dimensional SWE (2D-SWE) maps a two-dimensional stiffness field superimposed on the B-mode image in real time, allowing multiple ROIs and a color-scale representation of tissue elasticity. Three-dimensional SWE (3D-SWE) stiffness covers a complete volume, facilitating a more comprehensive analysis of tissue heterogeneities. SWE is supported by all major ultrasound manufacturers.

###### 5.2.1.2.3 Vibration-controlled transient elastography (VCTE)

Vibration-controlled transient elastography is a non-imaging technology that uses physical principles similar to those of SWE to measure tissue stiffness. It uses a probe-integrated mechanical driver instead of ARFI to generate shear waves. A simplified operator display guides the data acquisition, and internal algorithms assess the data quality. VCTE, commonly delivered by the FibroScan device (Echosens, Paris, France), measures the velocity of the shear wave that is converted to stiffness using the Young’s module. This which is directly proportional to tissue stiffness, serving as a surrogate marker for the degree of hepatic fibrosis (Castera et al., 2008). It is a rapid and non-invasive technique for the diagnosis and staging of liver fibrosis.

Although elastography is a valuable diagnostic tool, its integration into routine clinical practice remains constrained by the lack of universally validated cutoff values encompassing the entire spectrum of fibrosis stages (Duarte-Rojo et al., 2025). According to current international guidelines from the European Association for the Study of the Liver (EASL), threshold values of ≥8 kPa and ≥12 kPa are recommended for the identification of significant (F2) and advanced fibrosis (F3), respectively (Tacke et al., 2024). However, evidence from the broader literature indicates that alternative cutoff values can also be employed in clinical practice, reflecting variability in disease etiology and population (Tsochatzis et al., 2011; Pavlov et al., 2015; Duarte-Rojo et al., 2025).

An integrated feature of VCTE devices is the Controlled Attenuation Parameter (CAP), which quantifies hepatic steatosis by measuring the ultrasonic attenuation of the signal as it passes through the liver. CAP is the most widely clinically studied proprietary algorithm that has been available since 2010, by transient elastography (FibroScan®; EchoSens, Paris, France) using A-mode US to determine fat accumulation in liver tissue. CAP measures ultrasound attenuation at the central frequency of the VCTE at M or regular probe. A major increase in attenuation and a higher CAP value indicate a greater degree of fat accumulation, providing a point-of-care, semiquantitative assessment of both liver fat and fibrosis (Recio et al., 2013; Karlas et al., 2017; Rinella et al., 2023a; Rinella et al., 2023b). The accuracy of CAP in evaluating the degree of hepatic steatosis in patients with MASLD has been investigated in several patient populations. However, CAP only reflects the proportion of hepatocytes affected by steatosis, and it may not provide clues to the etiological factors in patients with fatty liver.

Consensus on definitive CAP thresholds for MASLD diagnosis is still being developed. Multiple cutoff values have been proposed and are being refined to enhance diagnostic accuracy. Distinct thresholds are often used to rule in or rule out hepatic steatosis (Petroff et al., 2021). Cutoff values commonly referenced in clinical practice are those outlined in the current MASLD management guidelines issued by the EASL. These guidelines specify CAP thresholds for grading hepatic steatosis: ≥248 dB/m for mild steatosis (S1), ≥268 dB/m for moderate steatosis (S2), and ≥280 dB/m for severe steatosis (S3) (Karlas et al., 2017; Tacke et al., 2024).

A recent meta-analysis encompassing 38 studies and 5,056 patients with MASLD demonstrated that CAP possesses high diagnostic performance, particularly for detecting any degree of steatosis (≥S1), with reported AUROC values of 0.95 for ≥S1, 0.84 for ≥S2, and 0.77 for ≥S3. However, CAP accuracy may be affected by factors such as obesity, ascites, advanced fibrosis, and the type of probe used (Wu et al., 2025). Moreover, its diagnostic capability is inferior to that of magnetic resonance imaging–derived proton density fat fraction (MRI-PDFF), and its performance declines with increasing body mass index compared to MRI-PDFF (Nogami et al., 2022).

Vibration-controlled transient elastography has been available for almost 20 years. Acoustic radiation force impulse (ARFI) techniques, including point SWE (pSWE) and two-dimensional (2D) SWE, have been available for almost 10 years. Consequently, multiple consensus guidelines have been developed. One of the most recent is from the Canadian Association of Radiologists (CAR) metabolic dysfunction-associated steatotic liver disease (MASLD) Working Group (WG), established in 2024 to provide unified practice recommendations for patients with MASLD. Their recommendations for MASLD screening in primary care are based on FIB-4 followed by imaging. They stated that transient elastography (TE), point shear wave elastography (pSWE), 2D shear wave elastography (2D-SWE), and magnetic resonance elastography (MRE) techniques provide similar diagnostic accuracy for the staging of liver fibrosis. While MRE may be the most accurate, the chosen method should be based on cost and availability (Wilson et al., 2025).

##### 5.2.1.3 Magnetic resonance imaging-proton density fat fraction (MRI-PDFF)

This advanced MRI technique measures the fraction of mobile protons in the liver attributable to liver fat (the PDFF), which is a direct measure of liver fat content. MRI-PDFF quantifies the liver fat fraction by measuring the ratio of the fat signal to the combined signal from water and fat protons in the tissue. It provides precise fat quantification in the liver; however, its routine use is limited by cost and accessibility. In MASLD, MRI-PDFF is the gold standard and an accurate imaging biomarker to 1) diagnose liver steatosis with a threshold of >5% liver fat, 2) monitor the progression of MASLD, and 3) assess treatment response by quantifying changes in liver fat content over time. Because MRI-PDFF is independent of acquisition parameters and agnostic to platform differences, it is a highly repeatable and reproducible biomarker (Malandris et al., 2024). A recent meta-analysis reported sensitivity and specificity values of 0.95 (95% CI, 0.89–0.98) and 0.98 (95% CI, 0.80–1.00), respectively, for distinguishing steatosis between S0 and S1–S3 when compared with liver biopsy (Wu et al., 2025). MRI-PDFF demonstrated superior diagnostic performance to CAP and was comparable or slightly better than AC in the detection of moderate and severe steatosis, with a strong correlation with histological grading (Wu et al., 2025). Nevertheless, the high cost and limited availability of MRI-PDFF restrict its widespread implementation as a routine screening tool in clinical practice.

### 5.3 Non-invasive methods for screening

Considerable efforts have focused on identifying acceptable, non-invasive, and cost-effective alternatives to liver biopsy. These approaches aim to improve the diagnosis and staging of hepatic steatosis and fibrosis in MASLD. To date, the best-validated non-invasive tests with the highest accuracy for ruling out advanced fibrosis are the FIB-4 and NAFLD fibrosis scores (Rinella et al., 2023a; Rinella et al., 2023b; Tacke et al., 2024). At present, international guidelines have not provided formal recommendations for a specific steatosis screening tool. Nevertheless, several are well established in research and epidemiological practice. The following section discusses the most prominent among them.

#### 5.3.1 Fatty liver index

The Fatty Liver Index (FLI) is a non-invasive screening algorithm, originally developed by Bedogni et al. (2006), to estimate the likelihood of hepatic steatosis in an Italian population (Bedogni et al., 2006). This index incorporates routinely measured biochemical and anthropometric parameters, specifically body mass index (BMI), waist circumference, triglycerides, and gamma-glutamyl transferase (GGT), into the following defined formula:

FLI=e^0.953*logetriglycerides+0.139*BMI+0.718*logeGGT+0.053*waist circumference−15.745 / 1+e^0.953*logetriglycerides+0.139*BMI+0.718*logeGGT+0.053*waist circumference−15.745 * 100

Validated against liver ultrasonography in Italian population, the FLI cutoff values are well established. An FLI > 60 indicates a high probability of hepatic steatosis (sensitivity 61%, specificity 86%). A value < 30 reflects low risk (sensitivity 87%, specificity 64%) (Bedogni et al., 2006). Using the same cutoffs in a South Korean population, the FLI showed comparable performance against MRI-PDFF and liver biopsy (sensitivity 85%, specificity 77%, AUROC 0.813) (Kim et al., 2023).

Subsequent studies have further evaluated the diagnostic accuracy of the FLI across diverse populations and against various reference standards, leading to the establishment of new population-specific cutoff values (Biciusca et al., 2023). A notable example is the study by Zhang et al. (2021), who utilized MRI-PDFF to diagnose hepatic steatosis in a Chinese cohort. Their analysis established an optimal cutoff value of ≥ 37.643, which yielded a sensitivity of 87.0% and specificity of 58.5% (Zhang et al., 2021). The variation in the optimal cutoff values across different cohorts highlights the importance of validating the FLI efficacy within specific populations to ensure its diagnostic precision.

When assessing the performance of the Fatty Liver Index (FLI) in discriminating between the severity levels of steatosis and steatohepatitis, it demonstrated strong diagnostic capability. In a South Korean population, the FLI yielded AUROC of 0.78 for distinguishing between patients with NAS of <3 and those with NAS of 3–4. Furthermore, it showed a higher AUROC of 0.845 for differentiating between patients with a NAS of 3-4 and those with a more severe score of >4 (Kim et al., 2023).

The FLI has demonstrated efficacy and accuracy in the management of MASLD and is routinely employed to screen and monitor disease progression in response to lifestyle changes (Biciusca et al., 2023; Kamari et al., 2023; Naomi et al., 2023; Trejo et al., 2025). However, its utility remains unvalidated in the Latin American population.

#### 5.3.2 Hepatic steatosis index

The Hepatic Steatosis Index (HSI) is derived from a linear combination of routinely available clinical and biochemical parameters and is calculated according to the following formula:

$$HSI=8\times \left(ALT/AST\;ratio\right)+Body\;Mass\;Index\;\left(BMI\right)+2\;\left(if\;female\right)+2\;\left(if\;Type\;2\;Diabetes\;is\;present\right)$$

HSI was developed and initially validated in a South Korean population. It showed strong diagnostic accuracy, with an AUROC of 0.812 (95% CI, 0.801–0.824), sensitivity of 91.3%, and specificity of 92.4%. An HSI value below 30 has a high negative predictive value, effectively ruling out significant steatosis. Conversely, an HSI value > 36 indicates a high probability of hepatic steatosis. Scores in the intermediate range of 30–36 are deemed indeterminate and are considered non-diagnostic, necessitating further evaluation (Lee et al., 2010).

Validation studies, using the cutoff values described above, have consistently confirmed the robust diagnostic performance of the HSI. When benchmarked against liver ultrasound, the HSI typically yields AUROC values exceeding 0.74 (Guo et al., 2023; Demirci and Sezer, 2025). These studies were conducted in populations from China and Turkey. Its performance is further corroborated in the South Korean population by comparison with the histological gold standard, liver biopsy, with one study reporting a high AUROC of 0.810 (87% sensitivity 74% specificity) (Kim et al., 2023). This diagnostic efficacy extends to diverse populations and methods. For instance, an evaluation within a Mexican cohort, using transient elastography as a reference, applied a cutoff value ≥40 for the detection of hepatic steatosis. Under this threshold, the HSI yielded an AUROC of 0.75 (0.66–0.83), with a sensitivity of 63.1%, and a specificity of 74.3% (Priego-Parra et al., 2024).

#### 5.3.3 Lipid Accumulation Product (LAP)

The Lipid Accumulation Product (LAP) is an index that integrates waist circumference and fasting triglyceride levels to estimate central lipid accumulation and its associated metabolic risk. It is widely employed as a simple and cost-effective marker for identifying individuals at risk for conditions such as metabolic syndrome, insulin resistance, cardiovascular disease, and arterial stiffness (Kahn, 2005; Xia et al., 2012; Nascimento-Ferreira et al., 2017; Shi et al., 2021; Ebrahimi et al., 2023; Papathanasiou et al., 2024). This index was calculated using the following formula:

$$For\;men:\left(waist\;circumference\;\left[cm\right]-65\right)\times triglyceride\;concentration\;\left[mmol/L\right]$$

$$For\;women:\left(waist\;circumference\;\left[cm\right]-58\right)\times triglyceride\;concentration\;\left[mmol/L\right]$$

Given the frequent comorbidity of metabolic disorders in individuals with MASLD, LAP has recently been evaluated as a tool for identifying those at risk. Research across diverse populations has consistently demonstrated strong diagnostic accuracy. A study using VCTE as a reference in U.S. adults established an optimal LAP cutoff of 47.67, which yielded a sensitivity of 79.2% and a specificity of 68.5% (Li H. et al., 2022). The same study identified sex-specific cutoffs of 46.43 (AUC: 0.818, 80.3% sensitivity, 68.8% specificity) for men and 47.84 (AUC: 0.808, 79% sensitivity, 67.7% specificity) for women. Similarly, a study in a Chinese cohort found LAP to be highly accurate, with AUC of 0.843 (95%CI 0.837, 0.849) for men and 0.887 (95% CI 0.882, 0.892) for women at lower cutoff values (30.5 and 23.0, respectively) (Dai et al., 2017). This high performance is corroborated by a Mexican study reporting an AUROC of 0.84 (95% IC 0.76–0.91, 75.4% sensitivity, 76.3% specificity, when applying a 53.4 cutoff) (Priego-Parra et al., 2024) and a meta-analysis that consolidated global data, finding a pooled sensitivity of 0.94 and a specificity of 0.85 for LAP as a screening tool for MASLD (Ebrahimi et al., 2023).

#### 5.3.4 KNAFLD score

The KNAFLD score is a non-invasive clinical prediction tool developed to assess the presence of hepatic steatosis. Unlike the previously mentioned instruments, it has also demonstrated utility in monitoring the progression of MASLD.

Originally developed and validated in Korea using the NAFLD Liver Fat Score as a diagnostic reference, the model employs cutoff values of <−3.285 to rule out steatosis and >0.884 to confirm its presence (Jeong et al., 2020). It was calculated using the following formula:

$$KNAFLD\;Score=0.913\times \left(sex;\;2\;if\;female,1\;if\;male\right)+0.089\times WC+0.032\times \left(systolic\;blood\;pressure+Fasting\;Serum\;Glucose\right)+0.007\times TG+0.105\times ALT-20.929$$

A subsequent validation study in China, which utilized liver biopsy as the diagnostic gold standard, reported a correlation coefficient of r = 0.52 for the score. When MRI-PDFF was used as the reference standard to evaluate its performance in predicting steatosis severity, the KNAFLD score showed strong diagnostic accuracy. It achieved an area under the curve (AUC) of 0.84 for predicting MASLD. Furthermore, it demonstrated a high predictive capacity for distinguishing mild steatosis-MASLD from controls (AUC = 0.77) and was effective in differentiating between mild and moderate-to-severe steatosis (AUC = 0.72, optimal cutoff = 4.13). The score also showed a high predictive value for assessing disease progression status, whether tow (Yang et al., 2024) worsening (AUC = 0.84) (Yang et al., 2024). To our knowledge, no study has evaluated the effectiveness of the KNAFLD score in a Latin American population.

#### 5.3.5 FIB-4 index

The FIB4 index was developed as a non-invasive panel to stage liver disease in patients with HIV-hepatitis C virus (HCV) co-infection. It relies on age, aspartate (AST) and aminotransferase (ALT) levels, and platelet count to estimate the risk of fibrosis. Since its development, this index has been validated with variable performance in subjects with HCV infection alone, HBV, NAFLD, and autoimmune hepatitis (Vallet-Pichard et al., 2007; Shah et al., 2009; Xu et al., 2022). The formula used to calculate the FIB-4 is as follows:

$$\left(Age\;\left[years\right]\;*\;AST\;\left[U/L\right]\right)\;/\;\left(Platelet\;count\;\left[10^{9}/L\right]\;*\surd ALT\;\left[U/L\right]\right)$$

According to the EASL–EASD–EASO Clinical Practice Guidelines, an FIB-4 index < 0.89 is categorized as low risk, while an FIB-4 index ≥ 2.67 is categorized as high risk of fibrosis. If the cutoffs are 0.89 < FIB-4 < 2.67, it is a gray zone, where further evaluation is required, with additional elastography or liver biopsy to identify the risk of fibrosis (Tacke et al., 2024).

FIB-4 index was principally developed by analyzing patients with an average age of 40 ± 7 years (Sterling et al., 2006). It has been reported that the appropriate cutoff for the FIB-4 index may vary by age because its formula includes this parameter (Ishiba et al., 2018). High FIB-4 index values were found predominantly in the elderly, and the effect of age was not negligible in those who underwent ultrasonography during health checkups. Therefore, it may be unsuitable as a primary screening tool for liver fibrosis in the general population, even though it can be easily calculated using routine biochemical data (Sugiyama et al., 2022).

#### 5.3.6 NAFLD fibrosis score

The NAFLD Fibrosis Score (NFS) is a well-established non-invasive tool used to estimate the risk of advanced liver fibrosis in patients with MASLD (Angulo et al., 2007; Tacke et al., 2024). This index integrates several independent predictors of fibrosis, namely, age, body mass index, the presence of hyperglycemia, platelet count, albumin level, and the AST/ALT ratio, into a defined formula as follows:

$$NFS=-1.675+\left(0.037\times age\right)+\left(0.094\times BMI\right)+\left(1.13\times impaired\;fasting\;glucose\;\left[yes=1,no=0\right]\right)+\left(0.99\times AST/ALT\;ratio\right)-\left(0.013\times platelet\;count\right)-\left(0.66\times albumin\right).$$

The clinical application of this score is guided by specific cutoffs according to current international guidelines. A score below −1.455 indicates a low risk, where advanced fibrosis (considered ≥F3 on the METAVIR scale) is unlikely; a score between −1.455 and 0.676 is indeterminate; and a score exceeding 0.676 signifies a high risk, where advanced fibrosis is likely (Tacke et al., 2024). The high-risk cutoff of 0.676 was initially validated with a high positive predictive value of 90% in the estimation cohort and 82% in the validation cohort (Angulo et al., 2007).

The prognostic utility of the NFS is supported by a systematic review that concluded that the score can effectively risk-stratify patients for liver-related morbidity and mortality, performing comparably to a liver biopsy (Lee et al., 2021). However, its diagnostic accuracy varies across populations. For instance, a European study that used liver biopsy as a reference found that the NFS had moderate accuracy, with an AUROC of 0.75 for predicting advanced fibrosis (≥F3) and an AUROC of 0.66 for predicting clinically significant fibrosis (≥F2) (Vali et al., 2023). This variability is further illustrated by a study conducted in Denmark. The NFS correctly classified only 54% of the study subjects. For significant fibrosis, it yielded an AUROC of 0.56 (95% CI, 0.46–0.65), with 88% sensitivity and 14% specificity. For advanced fibrosis, the AUROC was 0.66 (95% CI, 0.57–0.76), with 94% sensitivity and 16% specificity (Kjaergaard et al., 2023). These findings suggest that, although highly valuable for stratification, the precision of the NFS may vary across populations. To date, no formal validation has been conducted in Latin America.

#### 5.3.7 Limitations and scope of screening tools

Noninvasive screening tools serve a vital role as initial screening instruments, providing a cost-effective and accessible method for identifying individuals at elevated risk who may warrant subsequent confirmatory investigation (Rinella et al., 2023a; Rinella et al., 2023b; Tacke et al., 2024). Their principal utility lies in primary care and epidemiological settings, where they can optimize resource allocation by reducing unnecessary recourse to advanced diagnostics (Lee et al., 2021; Kjaergaard et al., 2023; Yang et al., 2024).

However, these tools have some limitations that must be acknowledged. Their diagnostic accuracy remains imperfect, often yielding indeterminate results, and their performance characteristics can vary significantly across populations with different demographic, anthropometric and comorbid profiles (Lee et al., 2021; Kjaergaard et al., 2023; Yang et al., 2024).

It is important to highlight that most of these tools have been developed and validated outside Latin America, primarily in China, Europe, and South Korea. Only a limited number of studies have evaluated their effectiveness and adapted cutoff values in the region, with evidence available exclusively from Mexico. This underscores the need for further research to establish context-specific validation and ensure applicability in local clinical practice.

Consequently, while indispensable for triage and initial risk stratification, these tools are complementary to, rather than a replacement for, comprehensive clinical assessments and advanced diagnostic modalities.

In Table 3, we summarize the following recommendations that could be the best fit for LATAM care systems. In primary care, initiating with clinical risk scores like FLI, HIS, LAP, KNAFLD, FIB 4, and NFS could screen the risk and if elevated or in presence of persistent ALT abnormalities, the addition of an ultrasound could lead to a correct screening in most of the patients. In specialty care, patients with ultrasound suggested steatosis/fibrosis could be test with a TE with CAP if available or to patent serum-based diagnosis algorithms like STEATOTest2, NASHTest2 or FibroTest to confirm the steatosis/fibrosis. The use of MRI PDFF can be assigned only for discordant or high stakes decisions (e.g., trial enrollment, unclear ultrasound/TE).

**TABLE 3:** Screening, diagnostic and management tools for the evaluation of MASLD and ALD.

Symptom	Tool/Biomarker	Uses
Steatosis screening	FLI, HSI, LAP, Ultrasound	Initial screening for all. Consider controlled attenuation parameter (CAP) if available
Alcohol-use quantification	Phosphatidylethanol (PEth)	Objective marker for moderate to heavy intake
Metabolic dysfunction assessment	Clinical criteria + TG, HDL-Chol Glu, BMI, BP, HOMA-IR, GGT	Identifies cardiometabolic risk contributors
Fibrosis risk stratification	FIB-4, VCTE, NAS	Adjust cutoffs upward in MASLD+ALD patients
Referrals and follow-up	Liver biopsy, MRI-PDFF	Reserve for indeterminate or high-risk cases

BMI, body mass index; BP, blood pressure; CAP, controlled attenuation parameter; FIB-4, fibrosis-4 index; FLI, fatty liver index; GGT, gamma-glutamyl transferase; Glu, glucose; HDL-Chol, high-density lipoprotein cholesterol; HOMA-IR, homeostasis model assessment of insulin resistance; HSI, hepatic steatosis index; LAP, lipid accumulation product; MRI-PDFF, magnetic resonance imaging–proton density fat fraction; NAS, NAFLD activity score; PEth, phosphatidylethanol; TG, triglycerides; VCTE, vibration-controlled transient elastography.

As an additional contribution to screening practices, Cumpin and colleagues proposed a noninvasive screening algorithm for MASLD, developed in accordance with major society guidelines and targeted case-finding strategies (e.g., T2DM). The algorithm incorporates lower thresholds for FIB-4, VCTE and MRE, specifically tailored for Hispanic/Latino patients (Cumpian et al., 2025).

### 5.4 Comorbidity of MALSD and ALD in Mexico and Latin America: implications for MASLD diagnostics

MASLD is defined as the presence of hepatic steatosis plus at least one cardiometabolic criterion and limited alcohol intake (<140 g/week for women, <210 g/week for men). Alcohol-related liver disease (ALD) represents liver injury from chronic excessive alcohol consumption (>350 g/week for women and > 420 g/week for men), with a separate spectrum ranging from steatosis to cirrhosis. MASLD and Alcohol-Related Liver Disease (ALD) frequently coexist and overlap, often in a condition known as Metabolic and Alcohol-Associated Liver Disease (MetALD), which involves both metabolic risk factors and moderate alcohol intake or in the form of MASLD + ALD (metabolic dysfunction plus harmful alcohol use) (Rinella et al., 2023a; Rinella et al., 2023b). This dual burden synergistically worsens the progression and severity of liver damage, increasing the risk of fibrosis, cirrhosis, and HCC compared to MASLD or ALD alone. Both conditions share significant comorbidities, such as obesity and cardiovascular disease, and managing them requires addressing both metabolic factors and alcohol use.

In Latin America and globally, the overlap is not merely semantic but has direct implications for diagnosis, prognosis, and care pathways. Alcohol consumption and metabolic dysfunction synergistically accelerate steatosis, inflammation, and fibrosis progression. Even moderate alcohol intake can potentiate metabolic injury, as describe since patients with MetALD have higher risks of cirrhosis, hepatocellular carcinoma, and cardiovascular disease compared to MASLD alone (Leal-Lassalle et al., 2025). One of the challenges is that self-reported alcohol intake underestimates exposure; therefore, diagnostic can be complex. Also, the overlap affects non-invasive fibrosis scores and complicates risk stratification. In LATAM, genetics and cultural background made heavy drinking and obesity prevalent risks. Structured algorithms are essential to avoid misclassification and ensure appropriate management. Here, we propose the following recommendations.

An initial assessment where alcohol intake can be screened using a validated tool like AUDIT-C questionary followed by the exploration of risk factors like BMI, DM2, dyslipidemias and hypertension. This in hand with a biochemical alcohol consumption biomarker like PEth (phosphatidylethanol); or CDT (carbohydrate-deficient transferrin) or EtG (ethyl glucuronide) as alcohol direct biomarkers. However, in LATAM these biochemicals are not widely available in public health systems, then the use of NFS, FLI or FIB-4 could be the first-line exams. But here attention must be paid since a common feature in alcohol abuse is a disproportionate elevation of AST and ALT that can inflate these markers. Then a repetition of the biochemical tests in controlled conditions of alcohol consumption or the use of alternative markers (like APRI) should be evaluated. As next step, an ultrasound to detect steatosis is recommended, followed by transient elastography with CAP using XL probe in obese patients for fibrosis staging.

Criteria for MASLD diagnosis include presence of metabolic dysfunction and alcohol intake below risk thresholds and PEth negative. MetALD would be detected if metabolic dysfunction plus alcohol intake above threshold are detected and PEth shows positive. While ALD assessment includes elevated self-reported alcohol intake and absence or minimal metabolic dysfunction.

This proposal also could lead to practical health policies: like PEth testing incorporation in routine screening in primary care settings and when AST > ALT predominance in disproportionate elevated in serum levels. Regarding managing, patients with MetALD should get treatment for alcohol cessation and metabolic alterations reduction.

This comorbidity is particularly common in regions with high metabolic risk driven by rising obesity and T2DM rates and unhealthy lifestyles, alongside culturally entrenched patterns of alcohol use, such as in LATAM. In Mexico, the prevalence of overlapping MASLD+ALD remains poorly characterized. Regional data on MASLD+ALD prevalence are scarce; extrapolation from global studies suggests that up to 15%–20% of MASLD cases may exceed alcohol thresholds, reclassifying them into the overlap subgroup. This comorbidity can complicate MASLD diagnosis when ultrasound is used (as in many LATAM reports) because the sensitivity for steatosis detection declines in advanced fibrosis and when alcohol-induced steatosis coexists, potentially delaying MASLD recognition and fibrosis assessment and leading to underdiagnosis or misclassification of fibrosis severity. These diagnostic challenges include FIB-4 cutoffs validated in MAFLD populations that may misclassify fibrosis stage in MASLD+ALD, as alcohol-related inflammation can elevate aminotransferases independent of fibrosis. Failure to account for alcohol’s confounding effect on non-invasive markers risks misclassification of MASLD and ALD diagnostic ambiguity in overlapping diseases must be overcome to ensure accurate case identification, fibrosis staging, and appropriate management pathways. Clinicians should maintain a high index of suspicion for metabolic dysfunction in patients with any degree of alcohol use, particularly in regions such as Mexico, where the dual burdens of obesity and hazardous drinking converge. Based on this information, we propose a diagnostic workflow for this comorbidity (Table 3).

Adopting this MASLD framework enables the inclusion of metabolic dysfunction cases beyond strict alcohol limits, but practical application requires the integration of alcohol biomarkers (e.g., PEth) and recalibration of elastography and serum-based scores. Further regional research is needed to adapt global recommendations to local epidemiological and cultural contexts.

## 6 Perspectives

In light of the current knowledge and regional challenges, several perspectives can guide research, clinical practice, and public policy statements on MASLD in Mexico and Latin America: Development and validation of simple, point-of-care risk scores that combine clinical, laboratory, and anthropometric parameters available in this demographic region. Embedding steatosis screening into routine health checks facilitates early detection, especially in resource-limited settings. In addition, regional multicenter studies across Hispanic populations should be conducted to tailor cutoffs for transient elastography, MRI-PDFF, and serum fibrosis biomarkers, considering genetic variants and environmental backgrounds to improve MASLD diagnostic accuracy and treatment. Personalized treatment algorithms may enhance efficacy and reduce adverse effects. Establishing a Latin American MASLD registry could create an impulse that captures standardized phenotypic, biochemical, imaging, and outcome data and attract funding for research in the region. This resource will enable comparative effectiveness research, identification of novel risk modifiers, and real-world evaluations of therapeutic interventions. Quantifying cost savings from prevented cirrhosis and hepatocellular carcinoma will bolster advocacy for investing in MASLD care. Collaboration with nutrition, education, and urban planning sectors is required to address obesogenic environments. An area of opportunity in the region is the promotion of policies that incentivize physical activity, regulate sugar-sweetened beverages (SSB), and improve access to affordable and nutrient-dense foods.

Regulation of SSB includes school availability restrictions, taxes, advertising limits, government procurement rules, and product labeling, and Latin America has generated some of the most robust evidence worldwide providing valuable lessons for other regions where implementation and evaluation remain less advance (Carriedo et al., 2021). Since 2019, 21 countries in the region had SSB excise taxes, but tax structures, coverage, and enforcement vary widely. Chile, Mexico, and Ecuador stand out for comprehensive, multi-pronged strategies, while most other countries have implemented fewer or less integrated measures (Sandoval et al., 2021; Sánchez-Ortiz et al., 2025).

Evidence shows that SSB taxes and wide-ranging regulations can reduce purchases and consumption. Mexico’s 10% SSB tax led to a 7.6% average reduction in purchases over 2 years, with the largest decreases among low-income households (Arantxa Cochero et al., 2017). Chile’s multi-component law that includes tax, labeling, marketing, and school ban resulted in a 23.7% decline in high-sugar beverage purchases (Taillie et al., 2020). Continued research is needed to optimize and expand these public health strategies.

The analysis of the cost of disease to model the economic impact of population-level screening, lifestyle interventions, and emerging pharmacotherapies. Develop regionally tailored training modules on MASLD pathophysiology, interpretation of non-invasive diagnostics, and lifestyle counseling. These should be integrated into medical and personal health curricula to ensure sustainable expertise. Pharmacogenetic and metabolomic profiling in diverse Latin American populations should be explored to identify responders to novel MASLD therapies. By pursuing these perspectives in parallel, research, clinical implementation, policy reform, and education, Latin America can transform the MASLD landscape from reactive diagnosis to proactive prevention and management of MASLD.

## 7 Conclusion

The growing burden of MASLD in Mexico and Latin America reflects the complex interplay of its high prevalence, significant complications, and unique regional drivers. Epidemiological global studies have revealed that up to 30% of adults in urban centers exhibit hepatic steatosis, with substantial subset progressing to fibrosis, cirrhosis, or hepatocellular carcinoma. These complications underscore the urgent need for targeted public health interventions to educate populations about the benefits of lifestyle changes and the need for robust surveillance systems for screening in primary care centers to prevent its development. Underlying this high disease burden are multifactorial risk determinants, including the rising prevalence of obesity in both adult and pediatric cohorts, T2DM, sedentary lifestyles, highly prevalent genetic predispositions in mestizo and Indigenous populations, and exacerbated socioeconomic disparities that limit access to healthy nutrition. Cultural factors, such as harmful levels of alcohol consumption, further exacerbate metabolic derangements, amplifying the risk of disease. Despite clear internationally issued clinical guidelines, substantial barriers hinder timely diagnosis, including limited awareness among primary care providers, overwhelmed health systems that limit available time for proper screening in at-risk populations, and scarce infrastructure for non-invasive imaging-based screening and diagnosis, creating bottlenecks. These challenges disproportionately affect rural and low-income communities, which account for a large proportion of the Mexican and LATAM populations, delaying the initiation of lifestyle modification and pharmacological therapies proven to alter the disease trajectory.

Advances in non-invasive modalities, such as vibration-controlled transient elastography, magnetic resonance elastography, and serum fibrosis panels, offer promising alternatives to biopsy for diagnosis and prognosis determination. When integrated thoughtfully, these tools can streamline risk stratification, minimize patient discomfort and optimize resource allocation. However, validation studies in Mexico and Latin American populations remain sparse, highlighting a critical research gap in this area. Collaborative efforts must focus on harmonizing epidemiological surveillance, bolstering clinician education, expanding access to validated imaging and biomarker-based diagnostic algorithms, and of course, the few drug-based available treatments. Latin America can overcome existing barriers and implement precision approaches to MASLD care by fostering regional networks for data sharing and capacity building. Such coordinated actions are essential to mitigate the rising tide of MASLD and improve outcomes across the region.
